# Conversion to laparotomy during laparoscopic hysterectomy: a meta-analysis of prevalence and key risk factors

**DOI:** 10.3389/fsurg.2025.1522022

**Published:** 2025-05-06

**Authors:** Qing Luo, Yan Wang, Xiaoyun Zhang

**Affiliations:** Department of Gynaecology and Obstetrics, Jiuquan People’s Hospital, Jiuquan, Gansu, China

**Keywords:** conversion, laparoscopic hysterectomy, meta-analysis, prevalence, risk factors

## Abstract

**Background:**

This meta-analysis aimed to estimate the prevalence and identify risk factors for conversion to laparotomy during laparoscopic hysterectomy (LH) for both benign and malignant gynecologic conditions.

**Methods:**

A comprehensive search of PubMed, Embase, and the Cochrane Library was conducted to identify studies published between January 2000 and September 2024. Eligible studies reported the prevalence and risk factors for conversion to laparotomy in patients undergoing LH. Studies were assessed for quality using the Newcastle-Ottawa Scale (NOS), and data were extracted on patient demographics, surgical details, and outcomes. A random-effects model was used to pool prevalence estimates and analyze risk factors. Heterogeneity was assessed using the I^2^ statistic, and publication bias was evaluated with funnel plots and Egger's test.

**Results:**

A total of 12 studies, encompassing 12,785 patients, were included. The pooled prevalence of conversion to laparotomy was 6% (95% CI, 5%–7%), with significant heterogeneity (*I*^2^ = 91.8%, *p* < 0.001). Conversion rates were higher in patients with malignant conditions (11%; 95% CI, 9%–14%) compared to benign conditions (5%; 95% CI, 4%–6%). Key risk factors included a history of adhesions (OR, 3.13; 95% CI, 1.91–5.11) and higher BMI (OR, 1.20; 95% CI, 1.08–1.34). Protective factors included surgeon experience (OR, 0.22; 95% CI, 0.08–0.59) and high surgeon volume (OR, 0.57; 95% CI, 0.34–0.94).

**Conclusions:**

Conversion to laparotomy occurs in approximately 6% of LH cases, particularly in patients with malignancy, a history of adhesions, or higher BMI. Surgeon expertise and case volume may reduce the risk, highlighting the importance of preoperative risk assessment.

## Introduction

Laparoscopic hysterectomy (LH) has become the standard of care for a wide range of gynecologic conditions due to its minimally invasive nature (1, 2). Compared with traditional open abdominal hysterectomy, LH was associated with reduced postoperative pain, shorter recovery times, less intraoperative blood loss, and fewer overall complications (3, 4). These advantages have led to its widespread adoption across various clinical settings. However, despite its benefits, a proportion of LH procedures require conversion to laparotomy due to intraoperative complications (5). Conversion not only undermines the advantages of LH but also increases morbidity, extends hospitalization, and delays recovery.

Conversion to laparotomy is typically driven by factors including excessive bleeding, dense pelvic adhesions, unanticipated large uterine size, or difficult anatomical visualization (5–7). The rates of conversion vary widely, with estimates ranging from less than 1% to over 10%, depending on patient populations, surgical expertise, and institutional practices (5, 6). This variability suggests that the true prevalence and risk factors associated with conversion remain poorly understood. Identifying patients at higher risk for conversion is essential to improve preoperative planning, enhance patient counseling, and optimize intraoperative decision-making.

Previous studies have suggested potential risk factors for conversion, including obesity, large uterine size, prior abdominal surgeries, and intraoperative complications (8, 9). However, the strength and consistency of these associations have not been systematically evaluated. The existing literature is fragmented, with varying definitions of conversion and inconsistent reporting of risk factors, making it difficult to form clear clinical guidelines for patient selection and management. Identifying modifiable and non-modifiable risk factors for LH conversion is essential for improving patient outcomes. By identifying high-risk patients preoperatively, surgeons can implement tailored strategies, such as enhanced preoperative imaging, alternative surgical techniques, or early decision-making to convert to laparotomy before complications arise.

The aim of this meta-analysis is to provide a comprehensive estimate of the prevalence of conversion to laparotomy in patients undergoing LH and to identify key risk factors associated with conversion. To date, no comprehensive meta-analysis has quantified the conversion rate during laparoscopic hysterectomy or systematically examined its risk factors across studies. By synthesizing data from diverse studies, this analysis seeks to offer clinicians a clearer evidence base for preoperative risk assessment and surgical planning, ultimately improving patient outcomes.

## Materials and methods

### Study design

This meta-analysis was conducted in accordance with the Preferred Reporting Items for Systematic Reviews and Meta-Analyses (PRISMA) guidelines to ensure transparency and methodological rigor (10). The primary objective of this study was to estimate the prevalence of conversion to laparotomy during LH and to identify the associated risk factors. We included studies that reported the prevalence and risk factors for conversion to laparotomy in patients undergoing LH. The study protocol was not registered.

### Search strategy

A comprehensive literature search was conducted across three electronic databases: PubMed, Embase, and the Cochrane Library. The search was performed using a combination of medical subject headings (MeSH) terms and free text related to “laparoscopic hysterectomy” “conversion to laparotomy” “risk factors”, “prevalence”, and their variants. The search was limited to studies published between January 2000 and September 2024 and restricted to articles in English. Additional studies were identified by manually searching the reference lists of the included articles. Unpublished data, conference abstracts, and grey literature were not considered for inclusion.

### Inclusion and exclusion criteria

We only included studies reporting both the prevalence and risk factors associated with conversion to laparotomy in patients undergoing LH for benign or malignant gynecologic conditions. The exclusion criteria included case reports, reviews, editorials, or studies without detailed data on conversion rates, studies involving procedures other than laparoscopic hysterectomy, and non-English language studies or those published before 2000.

### Data extraction

Two independent reviewers conducted data extraction using a pre-designed form. Discrepancies between reviewers were resolved by discussion or through consultation with a third reviewer. The following data were extracted from each included study, which including study characteristics (Authors, year of publication, country, study design, and sample size), patient demographics (Age, BMI, indication for surgery, uterine size, and history of prior abdominal surgeries), operative details (Duration of surgery, estimated blood loss, and the presence of intraoperative complications), and outcome measures (The prevalence of conversion to laparotomy and the reported risk factors associated with conversion). Generally, surgeon experience reflects either the surgeon's years of laparoscopic practice or the number of laparoscopic hysterectomies performed. Also, “high-volume” surgeons or centers are defined as those performing a large number of hysterectomies per year, whereas “low-volume” referred to those below that threshold. Considering that surgeon experience and surgeon volume may be associated with conversion to laparotomy, we also extracted data on their definition and cutoff value in included studies.

### Quality assessment

The methodological quality of the included studies was assessed using the Newcastle-Ottawa Scale (NOS) for comparative studies (11). The NOS assesses the risk of bias based on three domains: selection of study groups, comparability of groups, and ascertainment of outcomes. Studies were assigned scores ranging from 0 to 9, with scores ≥7 indicating high-quality studies. Quality assessment was independently performed by two reviewers, and discrepancies were resolved through consensus.

### Statistical analysis

The prevalence of conversion to laparotomy was pooled using a random-effects model to account for between-study heterogeneity (12, 13). Heterogeneity was assessed using the I^2^ statistic, with values >50% indicating significant heterogeneity (12, 13). Sensitivity analyses were performed through the “leave-one-out” method. To identify potential sources of heterogeneity, subgroup analyses were performed based on key study characteristics, including surgical indication, study design, sample size, NOS scores, and study center. Additionally, a meta-regression analysis involving sample size, publication year, and NOS scores were performed to explore potential sources of heterogeneity. For risk factor analysis, pooled odds ratios (ORs) and 95% confidence intervals (CIs) were calculated using a random-effects model. Only multivariate or adjusted risk factors reported in at least two included articles were considered for analysis. Publication bias was evaluated using funnel plots and Egger's test (8, 14). All statistical analyses were performed using Stata (version 12.0; StataCorp LLC). A *p*-value <0.05 was considered statistically significant.

## Results

### Study selection and characteristics

**Figure 1:**
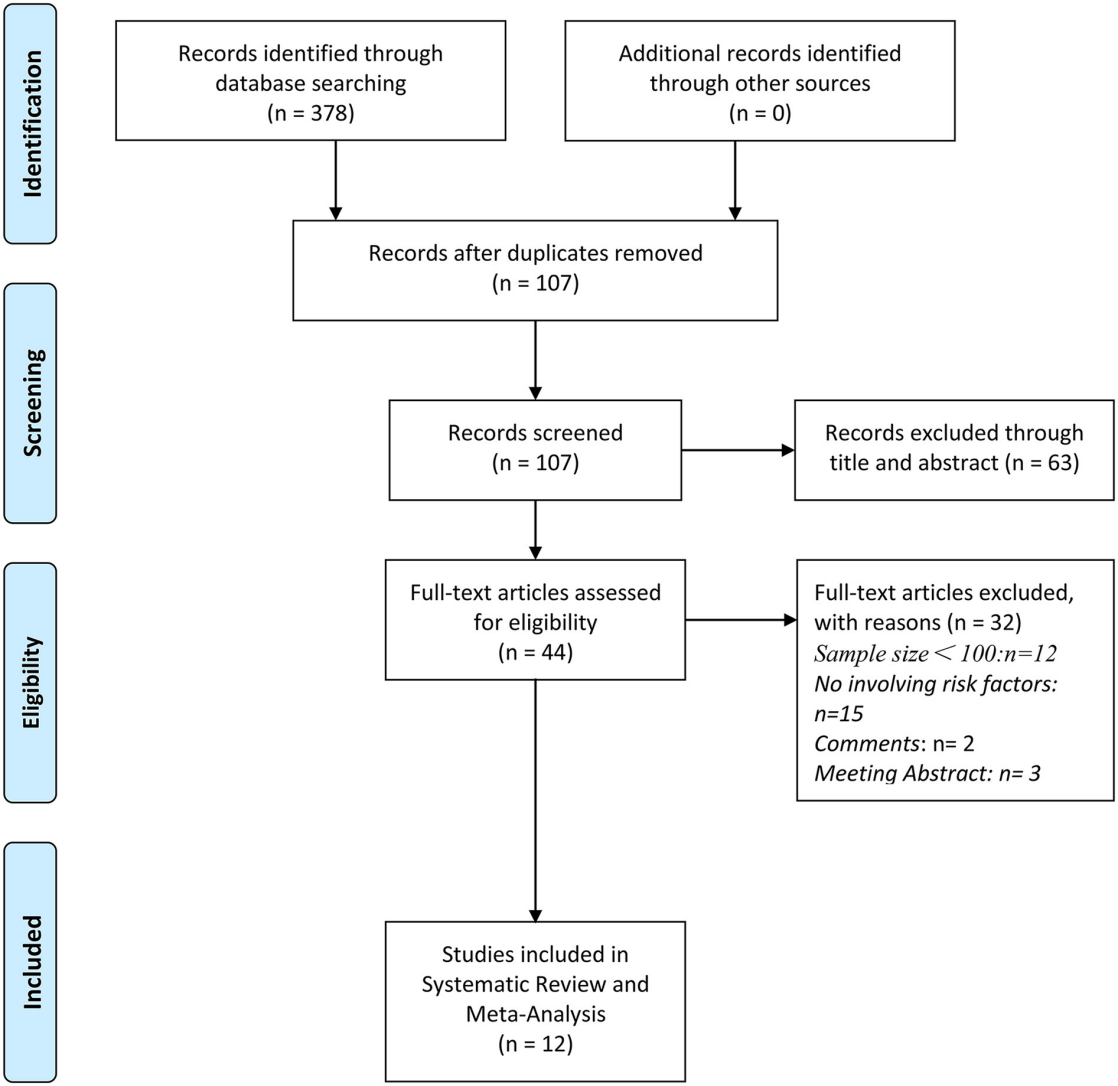
PRISMA flow diagram of study selection process.

A total of 12 studies were included in this meta-analysis after screening 378 records, following the removal of duplicates and exclusions (5–8, 15–22) (Figure 1). The key characteristics of these studies are detailed in Table 1. Most studies were retrospective cohort designs, published between 2005 and 2024, with study populations comprising women undergoing laparoscopic hysterectomy for benign gynecologic conditions, with some cases involving endometrial cancer. Conversion rates to laparotomy varied across studies, ranging from 3.93% to 12%. The majority of studies were single-center, with a few multi-center studies also included. The methodological quality of all the included studies was evaluated using the NOS scores (Table 2). Overall, 10 of the 12 studies received a score of 7 or higher, indicating generally high-quality methodologies.

**Table 1:** Baseline characteristics of studies on risk factors for conversion to laparotomy in patients undergoing laparoscopic hysterectomy.

Author (year)	Study design	Country	Enrollment period	Diagnosis	Age	Conversion to laparotomy rate	Study center
Franck 2005 (15)	Retrospective cohort study	France	Not specified	Benign gynecologic indications	47.9 ± 6.7	7% (29/416)	Single-center
Courtney 2016 (8)	Retrospective cohort study	USA	2013–2014	Benign gynecologic indications	Adult patients	3.93% (275/6,992)	Multi-center
Matsuo 2016 (18)	Retrospective cohort study	USA	2000–2014	Endometrial cancer	53.8 ± 10.3	12.0% (30/251)	Single-center
Bretschneider 2018 (19)	Retrospective cohort study	USA	2014–2016	Benign gynecologic indications	47.3 ± 6.1	5.5% (42/763)	Multi-center
Keurentjes 2018 (7)	Retrospective cohort study	Netherlands	2007–2010	Benign conditions and endometrial cancer	50.6 ± 12.0	5.0% (53/1,051)	Multi-center
Naveiro-Fuentes 2018 (20)	Retrospective cohort study	Spain	2008–2015	Benign and malignant gynecological diseases	50.8 ± 11.7	8.1% (19/236)	Single-center
Cianci 2019 (6)	Retrospective cohort study	Italy	2015–2017	Benign gynecologic indications	49 (Median)	9.8% (13/133)	Single-center
Brunes 2021 (20)	Population-based study	Sweden	2015–2017	Benign gynecological conditions	47 ± 10	5.7% (235/4,128)	Multi-center
Madhvani 2021 (22)	Retrospective cohort study	UK	2011–2018	Benign gynecological conditions	47.1 ± 9.7	6.0% (4,153/68,752)	Multi-center
Lamersdorf 2024 (5)	Retrospective cohort study	Germany	2016–2020	Benign gynecological conditions	48 (25–79)	7.3% (32/441)	Single-center
Claudia 2011 (16)	Randomised controlled trial	Netherlands	Not specified	Endometrial cancer	63 (39–89)	10.8% (20/185)	Multi-center
Markus 2013 (17)	Prospective cohort study	Germany	2003–2010	Benign gynecological conditions	47.5 ± 7.2	3.2% (62/1,952)	Single-center

**Table 2:** Quality evaluation of included studies (based on NOS criteria).

References	Is the case definition adequate?	Representativeness of the cases	Definition of Controls	Comparability of cases and controls based on the design or analysis	Ascertainment of exposure	Same method of ascertainment for cases and controls	Non response	Total scores
Franck 2005 (15)	*	*	*	*	*	*	0	6
Courtney 2016 (8)	*	*	*	**	*	*	*	8
Matsuo 2016 (18)	*	*	*	**	*	*	*	8
Bretschneider 2018 (19)	*	*	*	**	*	*	*	8
Keurentjes 2018 (7)	*	*	*	**	*	*	*	8
Naveiro-Fuentes 2018 (20)	*	*	*	*	*	*	*	8
Cianci 2019 (6)	*	*	*	*	*	*	0	7
Brunes 2021 (20)	*	*	*	**	*	*	*	8
Madhvani 2021 (22)	*	*	*	**	*	*	*	8
Lamersdorf 2024 (5)	*	*	*	**	*	*	0	7
Claudia 2011 (16)	*	*	*	*	*	*	*	7
Markus 2013 (17)	*	*	*	**	*	*	*	7

*One point. **Two point.

### Prevalence of conversion to laparotomy

**Figure 2:**
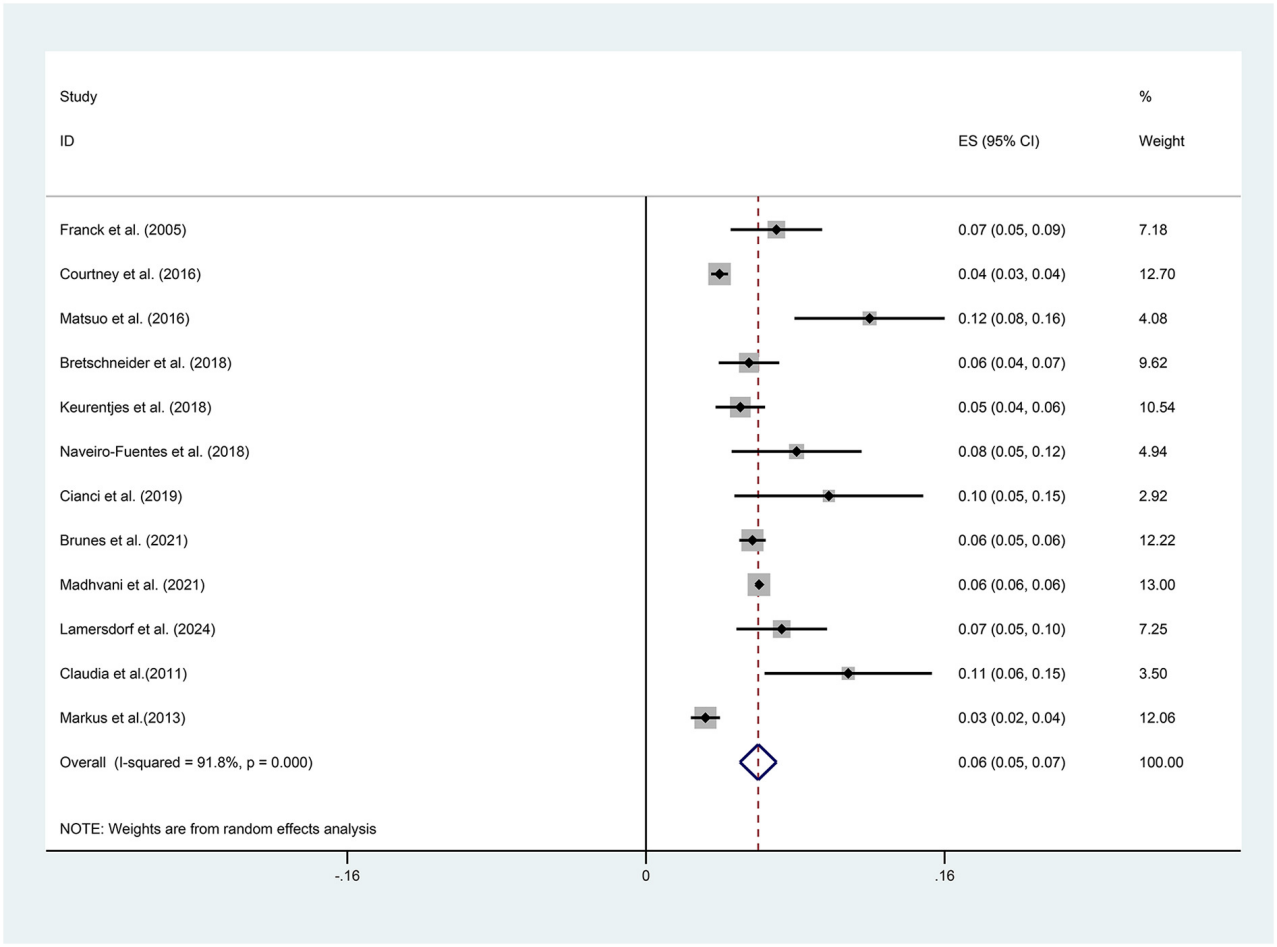
Forest plot of the prevalence of conversion to laparotomy during laparoscopic hysterectomy.

**Figure 3:**
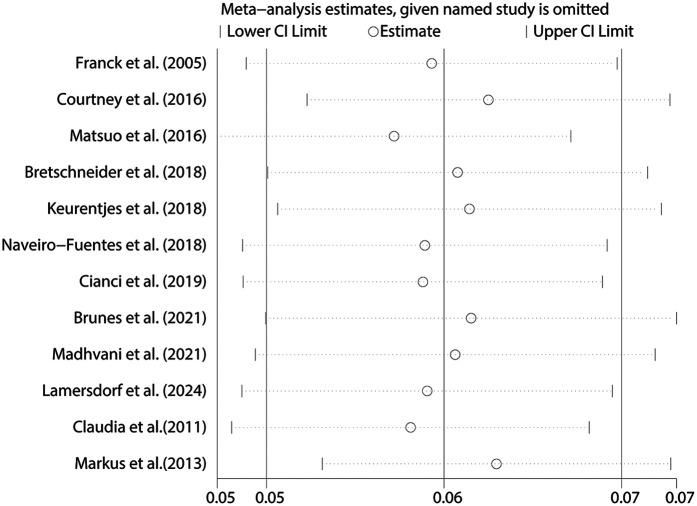
Sensitivity analysis for the pooled prevalence of conversion to laparotomy during laparoscopic hysterectomy.

**Figure 4:**
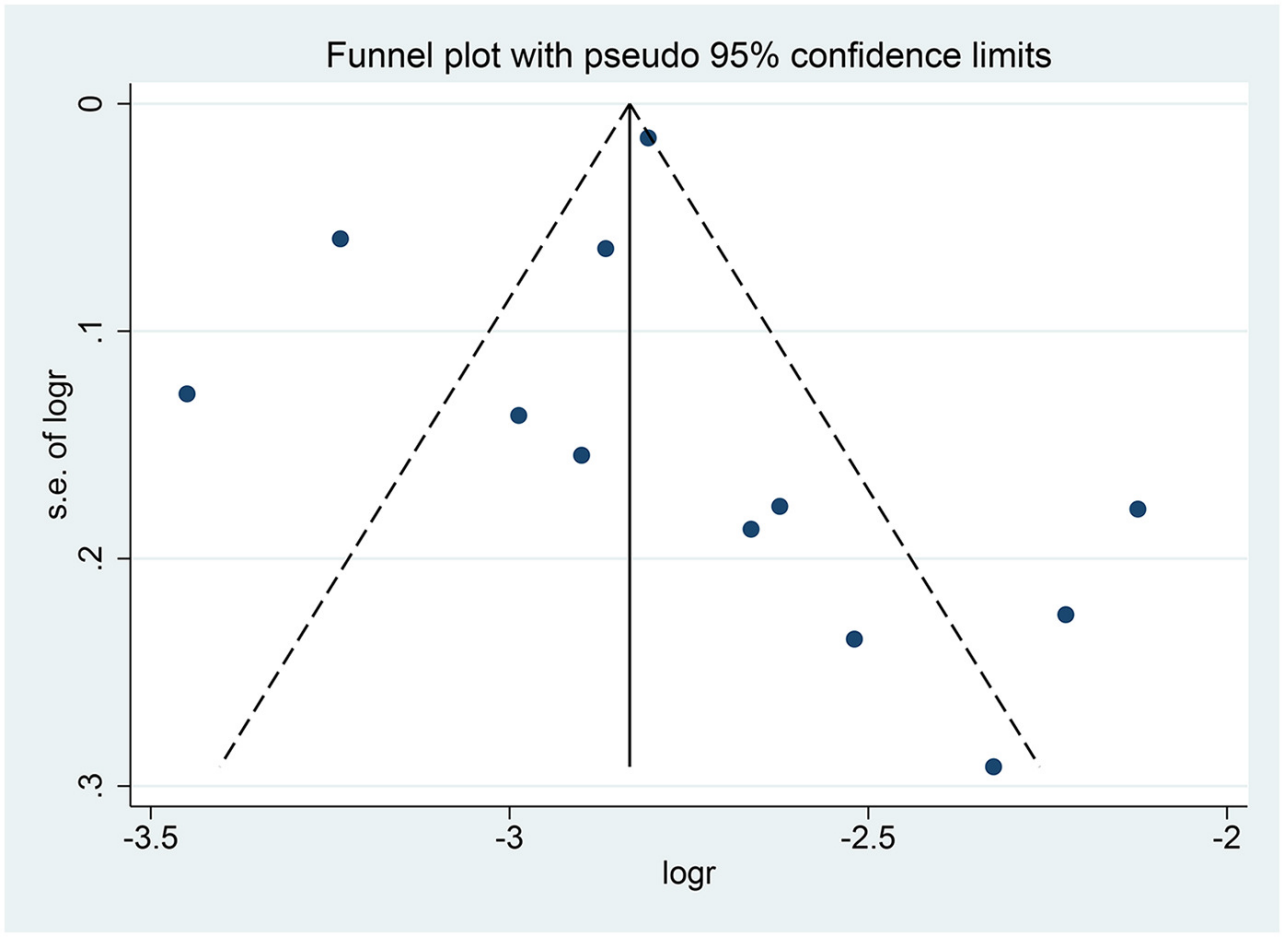
Funnel plot for assessing publication bias.

The pooled analysis demonstrated an overall conversion rate of 6% (95% CI, 5%–7%) among patients undergoing laparoscopic hysterectomy. Substantial heterogeneity was observed across studies (*I*^2^ = 91.8%, *p* < 0.001) (Figure 2). Sensitivity analysis revealed that no individual study disproportionately affected the overall estimate, confirming the stability and robustness of the findings (Figure 3). The funnel plot showed a possible symmetric distribution of studies, but statistical tests (Begg's Test: *P* = 0.086; Egger's test: *P* = 0.986) showed no significant publication bias (Figure 4).

### Subgroup analyses and meta-regression analysis

To investigate the sources of heterogeneity, subgroup analyses were performed based on study design, sample size, quality scores, surgical indications, and type of study center (Table 3). Retrospective studies reported a conversion rate of 6% (95% CI, 5%–7%), similar to the rate in prospective studies (7%; 95% CI, −0.01%–14%). Studies with larger sample sizes (greater than 1000 patients) demonstrated a slightly higher conversion rate (8%; 95% CI, 6%–10%) compared to studies with smaller sample sizes (5%; 95% CI, 4%–6%). Importantly, studies focused on malignant gynecologic conditions had a significantly higher conversion rate (11%; 95% CI, 9%–14%) compared to those involving benign conditions (5%; 95% CI, 4%–6%). Furthermore, to identify the source of heterogeneity across included studies, we used univariate meta-regression. Using sample size (*P* = 0.803), publication year (*P* = 0.795), and NOS scores (*P* = 0.698) as covariates revealed no significant associations.

**Table 3:** Subgroup analysis of the prevalence of conversion to laparotomy in patients undergoing laparoscopic hysterectomy.

Outcomes	Number of studies	OR (95% CI)	Heterogeneity I2 (%)
Pooled results	12	0.06 (0.05–0.07)	91.8
Subgroup analyses based on study design			
Retrospective studies	10	0.06 (0.05–0.07)	89.8
Prospective studies	2	0.07(-0.01–0.14)	90.8
Subgroup analyses based on number of sample size			
More than 1,000	7	0.08 (0.06–0.10)	55.1
Less than 1,000	5	0.05 (0.04–0.06)	96.5
Subgroup analyses based on quality of NOS scores			
No less than 8 points	7	0.06 (0.05–0.07)	92.8
Less than 8 points	5	0.07 (0.04–0.10)	87
Subgroup analyses based on different surgical indications			
Benign gynecological diseases	9	0.05 (0.04–0.06)	93.8
Malignant gynecological diseases	3	0.11 (0.09–0.14)	0
Subgroup analyses based on study center			
Single-center	6	0.08 (0.05–0.10)	87.9
Multi-center	6	0.05 (0.04–0.07)	93.5

### Risk factors for conversion

The multivariate meta-analysis identified key risk factors for conversion to laparotomy (Table 4). A history of adhesion was the strongest predictor, with a threefold increase in the risk of conversion (7 studies; OR, 3.127; 95% CI, 1.913–5.111; *p* < 0.001). Additionally, a higher BMI was associated with a significantly increased risk (6 studies; OR, 1.202; 95% CI, 1.082–1.335; *p* = 0.001). Only three studies in our review reported data on surgeon experience (6, 15, 20). In a pooled analysis of these studies, experienced surgeon had a lower conversion rate compared to low-volume surgeons (3 studies; OR, 0.22; 95% CI, 0.082–0.59; *p* = 0.003). Only four studies identified that surgeon volume may be potential risk factor for conversion to laparotomy (7, 8, 19, 21). The pooled estimate reveled that higher surgeon volume was similarly protective (4 studies; OR, 0.570; 95% CI, 0.344–0.943; *p* = 0.029).

**Table 4:** Multivariate meta-analysis for risk factors for conversion to laparotomy in patients undergoing laparoscopic hysterectomy.

Risk factors	Number of studies	Heterogeneity		OR (95% CI)	*P*
		*I*^2^ (%)	*P*		
BMI	6	83.8	0	1.202 (1.082–1.335)	0.001
Experienced surgeon	3	40.9	0.184	0.22 (0.082–0.59)	0.003
History of adhesion	7	84.1	0	3.127 (1.913–5.111)	0
Size of uterine lesions	3	77.1	0.013	2.274 (0.760–6.805)	0.142
Uterine weight	4	83.5	0	1.007 (0.992–1.021)	0.36
Cancer on final pathology	2	0	0.643	1.614 (0.957–2.722)	0.072
Surgeon volume	4	89.6	0	0.570 (0.344–0.943)	0.029
Age at surgery	4	68.4	0.024	1.008 (0.976–1.041)	0.621

Several factors, however, did not demonstrate statistically significant associations with the risk of conversion. These included the size of uterine lesions (3 studies; OR, 2.274; 95% CI, 0.760–6.805; *p* = 0.142), uterine weight (4 studies; OR, 1.007; 95% CI, 0.992–1.021; *p* = 0.36), cancer on final pathology (2 studies; OR, 1.614; 95% CI, 0.957–2.722; *p* = 0.072), and patient age at surgery (4 studies; OR, 1.008; 95% CI, 0.976–1.041; *p* = 0.621). Although these factors showed trends toward increased risk, they did not reach statistical significance.

## Discussion

This meta-analysis of 12 studies, encompassing both benign and malignant gynecologic conditions, provides a comprehensive overview of conversion rates from laparoscopic hysterectomy to laparotomy and identifies several key risk factors. The overall conversion rate was found to be 6%, with notable heterogeneity among the studies. Significant predictors of conversion included a history of adhesions and elevated BMI, while surgeon experience and volume were protective. These findings have important implications for clinical practice, particularly in patient selection, risk stratification, and surgical planning.

The overall conversion rate of 6% observed in this study aligns with previous reports, which suggest conversion rates between 3% and 15% depending on the complexity of the cases and surgical indications. For instance, a recent study found a conversion rate of 5.1% in a large retrospective study, which is consistent with the findings in this meta-analysis, especially in studies involving complex cases, such as malignancies (23). This highlights that despite advances in minimally invasive techniques, certain patient populations remain at high risk for conversion due to inherent procedural challenges.

The significantly higher conversion rates in cases involving malignant gynecologic conditions are also well-supported by existing literature (24). Malignancies often involve larger lesions, deeper tissue infiltration, and more extensive adhesions, all of which increase the complexity of laparoscopic surgery. The presence of such complications often necessitates conversion to laparotomy to ensure adequate oncologic outcomes and minimize intraoperative complications (24, 25). Thus, the findings of this meta-analysis corroborate previous evidence that conversion is more likely in cancer-related hysterectomies, underscoring the need for thorough preoperative planning and counseling in this patient group.

The identification of adhesions and BMI as major risk factors for conversion has direct clinical relevance. Adhesions, particularly from prior surgeries or conditions like endometriosis, obscure normal anatomic planes and make dissection more technically challenging. This not only increases operative time but also the likelihood of complications such as inadvertent organ injury. Our finding of a threefold increased risk of conversion in patients with adhesions is in line with prior research, which has consistently shown adhesions to be a major determinant of surgical difficulty in minimally invasive procedures (26). Surgeons should, therefore, consider preoperative imaging or diagnostic laparoscopy in patients with a known history of adhesions to better anticipate the risk of conversion and plan accordingly.

Higher BMI also emerged as a significant predictor of conversion, with an approximately 20% increased risk per unit increase in BMI. Obese patients are known to present unique challenges in laparoscopic surgery, including reduced visibility, limited instrument maneuverability, and increased operative time (27, 28). These factors collectively contribute to the higher likelihood of conversion to laparotomy in this population. Importantly, alternative surgical approaches, such as robotic-assisted laparoscopic hysterectomy, have been shown to mitigate some of these challenges in obese patients, suggesting that patient selection and surgical approach should be tailored based on individual risk factors (29). The protective effects of surgeon experience and volume on conversion rates highlight the critical role of surgical expertise in achieving optimal outcomes. Experienced surgeons are more adept at managing intraoperative complications and adapting to challenging surgical conditions, which likely explains their lower conversion rates. High-volume surgeons, in particular, have been shown to have superior outcomes across a range of surgical procedures due to their refined skills and familiarity with complex cases (30–32). Interestingly, our subgroup analysis revealed a trend that some larger multi-center studies reported higher overall conversion rates than smaller single-center studies, which seems to contradict the idea of increased surgeon volume decreasing conversion rate. This may be because large sample studies often include a wide range of surgeons and institutions, capturing variability in skill levels and case complexity. In contrast, when examining individual surgeon performance, our results indicate that surgeons with high case volumes tend to have lower conversion rates than those with lower volumes. In other words, a high-volume surgeon's expertise can mitigate conversion risk, even though studies that aggregate many surgeons of varying experience may show a higher average conversion rate. Therefore, these finding reinforces the argument for centralizing complex laparoscopic surgeries in high-volume centers to ensure that patients benefit from the expertise of experienced surgeons, thus reducing the risk of conversion and improving overall outcomes. Increasing evidence indicated that emerging surgical techniques, particularly robotic-assisted laparoscopic hysterectomy, have demonstrated potential in reducing conversion rates in high-risk patient populations. Robotic systems enhance visualization, dexterity, and precision, addressing common challenges posed by obesity and adhesions. Recent literature highlights the advantages of robotic approaches, suggesting potential benefits in managing complex cases (33, 34). Additionally, conditions such as extensive adhesions or malignancies are well-established to elevate conversion risks significantly (35). Technological advancements may particularly benefit these challenging patient groups by reducing procedural complexity and enhancing surgical outcomes.

Despite the strengths of this meta-analysis, including a large pooled sample size and the identification of significant risk factors, several limitations must be acknowledged. Firstly, we observed significant statistical heterogeneity across included studies. Despite performing subgroup analyses and meta-regression analysis, we did not identify potential sources of heterogeneity. This suggests that variability may originate from factors not sufficiently captured by the available data, possibly involving variations in surgical training, perioperative management protocols, and reporting standards across institutions. Nevertheless, our sensitivity analyses demonstrated stability and consistency in the pooled results, indicating that the observed heterogeneity did not materially undermine the validity of our conclusions. Future prospective studies adopting standardized outcome definitions, uniform reporting guidelines, and detailed documentation of surgical experience and institutional protocols may better clarify these sources of variability and further enhance the precision of subsequent meta-analyses. Additionally, the predominance of retrospective studies introduces selection and reporting biases, so the current results should be interpreted with caution. We explicitly highlight these limitations and strongly advocate for prospective, multicenter studies to establish more definitive evidence regarding the predictors of conversion, enhancing the validity and generalizability of these findings. Another limitation is the inconsistent reporting of certain variables, such as the extent of adhesions or specific thresholds for BMI, which may have led to an underestimation or overestimation of their impact on conversion. Standardization of reporting in future studies would enhance the reliability of risk stratification. Also, only limited studies reported risk factors, including surgeon experience and volume in our meta-analysis, which limits the strength of conclusions about these factors. The meta-analysis for these variables was based on a small sample and should be interpreted cautiously. For instance, several variables, including uterine size and uterine weight, did not reach statistical significance. The lack of statistical significance in these variables warrants cautious interpretation. Specifically, the relatively wide confidence intervals associated with uterine size suggest the presence of potential clinical relevance, which may not have been adequately captured due to insufficient statistical power or variability in the measurement criteria across included studies. Moreover, inconsistency in defining thresholds for uterine size or weight, as well as varying degrees of precision in measurement and reporting, may have contributed to this non-significance. Similarly, only three or four studies reported data on surgical experience or volume and pooled estimate indicated that experienced surgeon or higher surgeon volume was associated with a lower conversion rate. However, due to the limited data, any findings related to surgeon experience and volume should be interpreted as preliminary. Therefore, the absence of a statistically significant association in our meta-analysis should not be interpreted as definitive evidence against their clinical importance. Rather, it underscores the necessity for further investigation through larger, well-designed prospective studies.

## Conclusions

This meta-analysis provides important evidence regarding the prevalence of conversion to laparotomy during laparoscopic hysterectomy and identifies key risk factors such as adhesions and BMI. The possible protective effects of surgeon experience and case volume further underscore the importance of surgical expertise in reducing conversion rates. These findings have important clinical implications for the preoperative assessment, surgical planning, and centralization of complex cases. Future research, particularly prospective and randomized studies, is needed to refine risk prediction models and validate these findings in broader populations.

## Data Availability

The original contributions presented in the study are included in the article/Supplementary Material, further inquiries can be directed to the corresponding author.
